# Challenges and advanced concepts for the assessment of learning and memory function in mice

**DOI:** 10.3389/fnbeh.2023.1230082

**Published:** 2023-09-21

**Authors:** Benjamin Lang, Pia Kahnau, Katharina Hohlbaum, Paul Mieske, Niek P. Andresen, Marcus N. Boon, Christa Thöne-Reineke, Lars Lewejohann, Kai Diederich

**Affiliations:** ^1^Animal Behavior and Laboratory Animal Science, Department of Veterinary Medicine, Institute for Animal Welfare, Free University of Berlin, Berlin, Germany; ^2^Science of Intelligence, Research Cluster of Excellence, Berlin, Germany; ^3^Federal Institute for Risk Assessment (BfR), Berlin, Germany; ^4^Computer Vision and Remote Sensing, Technical University Berlin, Berlin, Germany; ^5^Modeling of Cognitive Processes, Technical University of Berlin, Berlin, Germany

**Keywords:** learning and memory, animal cognition, home cage analysis, social interaction, automated tracking, ecological validity, animal welfare

## Abstract

The mechanisms underlying the formation and retrieval of memories are still an active area of research and discussion. Manifold models have been proposed and refined over the years, with most assuming a dichotomy between memory processes involving non-conscious and conscious mechanisms. Despite our incomplete understanding of the underlying mechanisms, tests of memory and learning count among the most performed behavioral experiments. Here, we will discuss available protocols for testing learning and memory using the example of the most prevalent animal species in research, the laboratory mouse. A wide range of protocols has been developed in mice to test, e.g., object recognition, spatial learning, procedural memory, sequential problem solving, operant- and fear conditioning, and social recognition. Those assays are carried out with individual subjects in apparatuses such as arenas and mazes, which allow for a high degree of standardization across laboratories and straightforward data interpretation but are not without caveats and limitations. In animal research, there is growing concern about the translatability of study results and animal welfare, leading to novel approaches beyond established protocols. Here, we present some of the more recent developments and more advanced concepts in learning and memory testing, such as multi-step sequential lockboxes, assays involving groups of animals, as well as home cage-based assays supported by automated tracking solutions; and weight their potential and limitations against those of established paradigms. Shifting the focus of learning tests from the classical experimental chamber to settings which are more natural for rodents comes with a new set of challenges for behavioral researchers, but also offers the opportunity to understand memory formation and retrieval in a more conclusive way than has been attainable with conventional test protocols. We predict and embrace an increase in studies relying on methods involving a higher degree of automatization, more naturalistic- and home cage-based experimental setting as well as more integrated learning tasks in the future. We are confident these trends are suited to alleviate the burden on animal subjects and improve study designs in memory research.

## Introduction

In recent years, we ascribe the label ‘artificial intelligence’ to machine learning algorithms deployed in applications such as language models, image generation, or autonomous vehicles with increasing generosity. While ‘artificial intelligence’ saw a spillover from research applications and tech industries into the public awareness, we still understand surprisingly little about what constitutes intelligent behavior. In a very general scheme, higher-order cognitive and memory functions form the basis for the temporal coordination of actions to achieve a specific goal. As a result, complex sequences of behaviors can be performed using ordered priorities according to goals and subgoals which can be described as insightful behavior (Reznikova, 2007).

**Figure 1:**
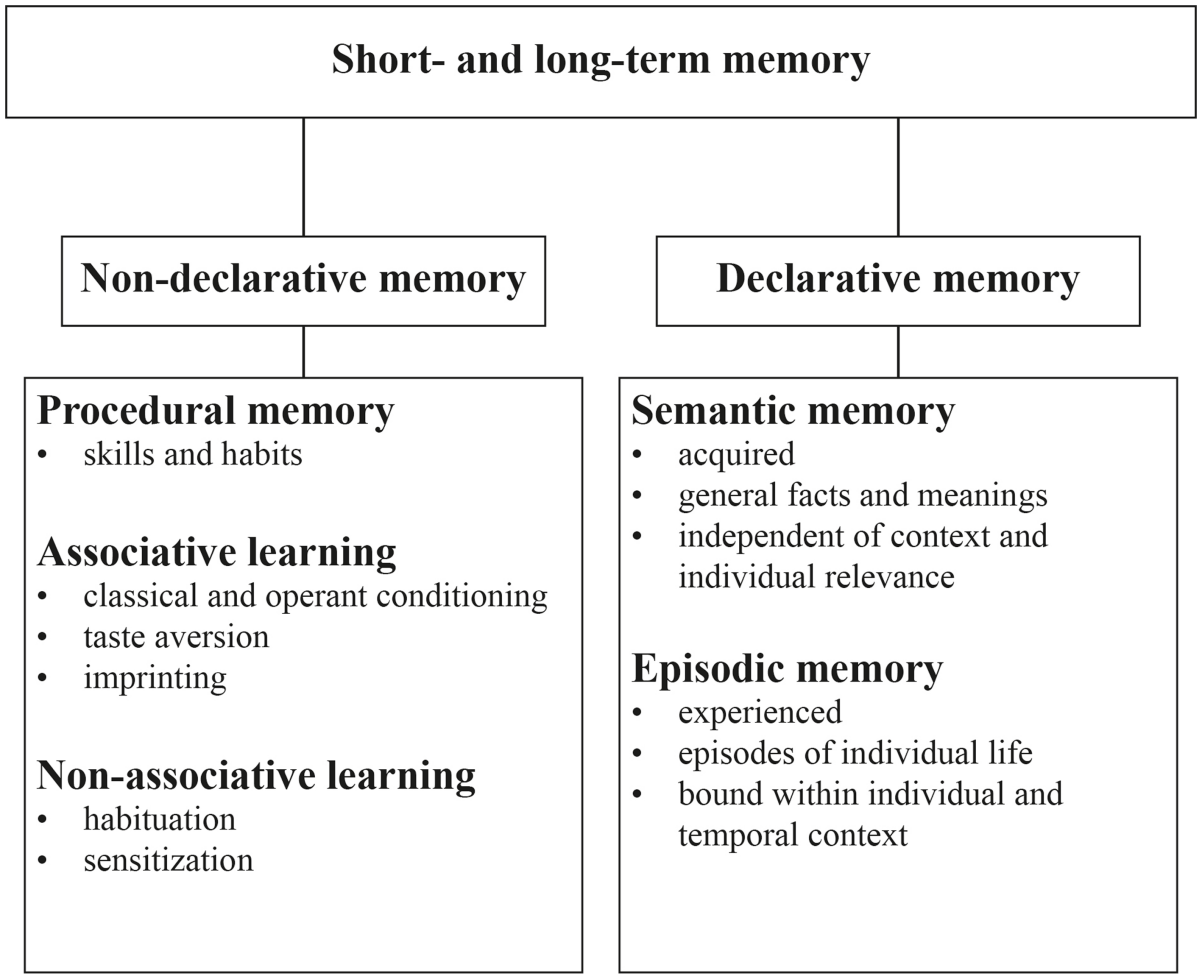
Classification of the different types of memory processes (adapted from Brem et al., 2013).

In non-human animals, however, insightful behavior is notoriously difficult to measure. Instead, animal studies often attempt to construct simplified tests so that specific memory components (e.g., spatial memory in mazes) can be quantified. Conventional behavioral tests measure specific motor responses or their suppression as qualitative indicators of a memory trace. Quantifying motor responses to measure the strength of memory may be confounded by a variety of underlying mechanisms that are difficult to dissect and could therefore lead to inconclusive results (Lipp and Wolfer, 2022). Given the complexity in which intelligent behavior is seen, it stands to reason that there is probably only weak decomposability regarding the common schemes of memory systems involved. For example, higher order memory functions such as episodic memory are usually attributed to declarative memory (Figure 1) but certainly also rely to a large degree on procedural memory such as classical and operant conditioning as well as non-associative memory.

Despite those inherent challenges, memory and learning abilities are among the most tested behavioral parameters relevant in basic research, drug development, and biomedical research in disease models. Standardized tests have been developed to assess the function of specific brain regions and subcategories of memory. Rodents, particularly mice, are by far the most used animal species in research (Hickman et al., 2017). Their popularity results from reasonably genetic similarity to humans (Pennacchio, 2003) short generation times, and comparatively low demands on housing and maintenance (Scharmann, 1991; Deacon, 2006). Finally, the vast body of preexisting research available on them encourages the continuous usage of mice as model species in a positive feedback loop.

A wide selection of assays for the quantification of memory subtypes have been either adapted from other species or specifically developed for mice (e.g., Vorhees and Williams, 2006; Leger et al., 2013). These assays may be categorized along the specific memory functions they address, such as semantic-, episodic-like-, or associative memory (Table 1). Due to the reductive nature of such tests of memory subdomains, they are performed in a temporally and spatially confined manner. Usually, this takes place outside the animals’ familiar environment in simple experimental apparatuses such as mazes or skinner boxes. The prevalence of such test protocols in mice allows for a comparatively high degree of standardization while maintaining low financial and technological thresholds. However, they are inherently limited in the scope of insight they can grant into memory processes and further may also pose avoidable stress on the animals (Hölscher, 1999; Harrison et al., 2009).

**Table 1:** Behavioral tests across cognitive domains and corresponding structural correlates.

Cognitive domain	Behavioral test	Main structural correlates	Example references
Semantic memory	Object recognition, (non)matching-to sample, reversal learning, contextual fear conditioning	Prefrontal cortex, hippocampus, perirhinal cortex, visual cortex	Ennaceur and Delacour (1988), Curzon et al. (2009), Leger et al. (2013), and Goltstein et al. (2021)
Episodic-like memory	Delayed-(non)matching-to-sample task, What-Where-When (Which) task, spatial navigation	Hippocampus, prefrontal cortex, amygdala	Barnes (1979), Morris (1981, 2001), Robinson and Crawley (1993), Vorhees and Williams (2006), DeVito and Eichenbaum (2010), and Davis et al. (2013)
Associative learning/memory	Passive avoidance, active avoidance, fear conditioning, classical and operant conditioning	Hippocampus, prefrontal cortex, frontal motor areas, striatum	Van der Poel (1967), Schleyer et al. (2018), and Van Gurp et al. (2020)
Procedural memory	Motor-skill-learning, operant conditioning, procedural T-maze task	Cerebellum, striatum	Dere et al. (2008), Cayzac et al. (2011), and Willis et al. (2017)
Executive function	Set-shifting tasks, reversal learning, Go/No-go task, delayed alternation, prepulse inhibition, five-choice serial reaction time task	Prefrontal cortex, basal ganglia, amygdala	Kesner and Churchwell (2011), Bizon et al. (2012), Endo et al. (2012), Mar et al. (2013), Shepard et al. (2017), and Sable et al. (2021)

In recent years, new technologies and refined methodologies have paved the way for new experimental approaches that have enabled a better understanding of the mechanisms underlying memory. However, novel approaches also come about with new challenges for the researchers. Recent developments, as well as the limitations and unique advantages of past and present testing methods, are discussed in this review.

### Types of memory

Memory functions have been categorized along various dimensions including temporal dimension (James, 1890), content, or acquisition mechanisms (Brem et al., 2013). In the 1980s, a categorization based on distinct neural structures involved in different memory functions was brought forward by Squire (1986). The dichotomy which introduced ‘declarative’ versus ‘nondeclarative’ memory, is still relevant to modern models of memory and will also largely provide the framework for the present review. As the classification of memory types is based primarily on observations in humans and other primates, the extent to which such an anthropocentric approach is appropriate for classifying memory functions in mice is a matter for discussion. Of note, neither competing dichotomies nor the assigned subcategories are mutually exclusive. Rather, there appears to be overlap between memory functions and the underlying involved neural structures, and modern models assume a plastic and dynamic structure (Brem et al., 2013).

### Non-declarative memory

Non-declarative memory is non-consciously accessible and thus can be expressed without conscious retrieval of information from memory. It is also referred to as implicit memory and is highly involved in the acquisition and use of motor skills, habits, and emotional responses. These skills are acquired through practice and repetition, and once learned, they can be performed without conscious awareness (Squire and Zola, 1996).

Procedural memory is a type of non-declarative memory that involves the acquisition and retention of specific procedures or skills. Procedural memory is characterized by its automaticity and its resistance to forgetting over time, as the memory of the task becomes increasingly ingrained through practice and repetition. Different types of procedural memories are sometimes referred to separately as motor, perceptual, or cognitive procedural memories (Zichlin, 2011).

Associative learning refers to the process by which an organism learns to associate unrelated stimuli to form a stimulus–response pair resulting in a distinct behavior. It involves linking two or more events that occur together, such that the occurrence of one event reliably predicts the occurrence of the other (Jozefowiez, 2012).

Non-associative learning refers to a type of learning in which an organism’s behavior is changed in response to a single stimulus, without the need for pairing that stimulus with another stimulus. An organism’s behavioral changes in response to a stimulus over time would present an instance of non-associative learning (Ioannou and Anastassiou-Hadjicharalambous, 2018). There are two types of non-associative learning: habituation and sensitization. Habituation occurs when an organism reduces its response to a stimulus after repeated exposure to it, while sensitization occurs when an organism increases its response to a stimulus (Ioannou and Anastassiou-Hadjicharalambous, 2018).

#### Non-associative learning in animals

Habituation and sensitization are important adaptive responses that allow animals to modulate their behavioral responses to different stimuli in their environment. They have been observed in a wide variety of animal species, from invertebrates to mammals including humans (Byrne and Hawkins, 2015). Habituation is the process of diminishing a behavioral response to a stimulus that is presented repeatedly. A classic example is the gill-withdrawal reflex in *Aplysia* (Hawkins et al., 1989). Sensitization on the other hand is the process of increasing a behavioral response or lowering the threshold to elicit the response. For example, *Aplysia*’s gill-withdrawal reflex is enhanced after a single electric shock is delivered to the snail’s tail (Hawkins et al., 1989).

#### Associative learning in animals

Many animals, including vertebrates as well as invertebrates, are capable of associative learning. Classical and operant conditioning are the most common forms of associative learning. Classical conditioning is well known from the experiments of Ivan P. Pavlov showing that an unconditioned response (e.g., a dog salivating when seeing food) is paired with a formerly neutral stimulus (e.g., ringing a bell) when repeatedly presented in close association with an unconditioned stimulus (e.g., the food). Thereby the neutral stimulus becomes a conditioned stimulus which similarly to the unconditioned stimulus is capable of eliciting the response e.g., salivating when hearing a bell without food being present (Pavlov, 1897). In operant conditioning, a behavior is associated with a reward or a punishment and, by this association, the frequency of showing the behavior is either increased or decreased. The concept of operant conditioning dates back to the work of Edward L. Thorndike formulating the “law of effect” (Thorndike, 1898) and describes extensive studies on associative learning in cats, dogs, and chicken. Later, Skinner mastered operant conditioning using test boxes (“Skinner box”) where various species of animals were presented with an operandum (e.g., a lever or a disk to peck on) which was associated with rewards or punishments (Skinner, 1935, 1938).

A very basal form of associative learning quite similar to classical conditioning is conditioned taste aversion also known as the “Sauce Bearnaise syndrome.” The differences to classical conditioning are that usually a single presentation of the neutral stimulus is sufficient and that a considerable delay can be between the stimulus presentation and the unconditioned reaction e.g., associating a foul taste with Sauce Bearnaise due to feeling nausea hours after eating a Filet Mignon that was served with the sauce (Seligman and Hager, 1972; Stensmyr and Caron, 2020).

Imprinting is a form of learning where during a sensitive phase an association between a stimulus and a behavioral response is formed. Filial and sexual imprinting have been studied mainly in birds (Hess, 1958; Lorenz, 1958; Immelmann, 1972; Bateson, 1978). Although imprinting is known to be present in mammals as well, it has rarely been studied in the context of learning and memory in rodents.

Associative learning and especially classical- and operant conditioning are the most common techniques used in training animals. Dogs are capable of learning associations between certain sounds and commands, and between certain behaviors and rewards or punishments. Aside from human animal interactions there are many examples of associative learning under natural conditions all over the taxa within the animal kingdom. Many species of birds are capable of learning to associate specific sounds or visual cues with food, predators, or other relevant stimuli. Fish are capable of learning associations between specific smells or sounds and food, as well as between specific behaviors and rewards or punishments. Insects are capable of learning associations between specific colors or shapes and food sources. Octopuses and squids are capable of learning associations between specific objects or stimuli and rewards or punishments. Associative learning is shown in various contexts, and it is a crucial mechanism for animals to adapt to their environments (Dickinson, 2012).

### Declarative memory

Declarative memory involves recollection of factual information, such as events, places, or concepts (Squire, 1992). It is also called explicit memory because, at least in humans, it involves the conscious and intentional retrieval of memory content.

Declarative memory can be further divided into two subtypes. One is semantic memory, which is the memory of general knowledge and concepts that are not tied to a specific event or experience. Secondly, episodic memory is the recalling of specific events or personal experiences that are tied to a particular time and place.

#### Semantic memory in animals

Semantic memory is a type of memory that stores general knowledge about the world, including concepts, facts, and meanings of words. It refers to the ability to recall and understand information about the world, such as the names of countries, historical events, common objects, and their attributes (Tulving, 1972).

Following this definition, it is obvious that non-human animals do not have the same capacity for semantic memory as humans. Especially with regard to the naming of outer world objects and thus forming an explicit internal representation of the outer world. However, studies have shown that some non-human animals can learn and recognize symbols and understand their meaning, suggesting that the basic concept of semantic memory can also be applied to some non-human animals. For example, chimpanzees have been shown to be able to learn the meanings of words and use them appropriately in new situations (Slocombe and Zuberbühler, 2005; Clay and Zuberbühler, 2009; Gabrić, 2022).

Furthermore, there is accumulating evidence that mice may also have some form of semantic memory. Research has shown that mice have the ability to learn and remember locations of objects in their environment (Romberg et al., 2018; Smith et al., 2018) and the identities of conspecifics (Kogan et al., 2000) indicating that they are capable of forming and retaining semantic memories. A recent study found that mice are able to discriminate, generalize, and remember visual categories, and found that these perceptual and semantic features of learned category associations are already represented in the visual cortex (Goltstein et al., 2021).

The neural basis of semantic memory is complex and involves multiple brain regions. The medial prefrontal cortex and the hippocampus have been identified to be involved in encoding and retrieval of different types of semantic memories across various species of animals, including mice and humans. While the complexity and extent of semantic memory in mice may be limited compared to that of humans, it would not be farfetched to assume that mice also have a basic form of semantic memory.

#### Episodic memory in animals

Episodic memory is a type of long-term memory that allows individuals to recall specific events or experiences that occurred at a particular time and place in their lives. It involves the ability to remember details such as sensory information, emotions, and contextual information surrounding an event (Tulving, 1972; Tulving and Markowitsch, 1998; Ferbinteanu et al., 2006).

While some animals exhibit advanced cognitive abilities there is currently no consensus as to whether non-human animals possess true episodic memory (Hampton and Schwartz, 2004; Dere et al., 2006). There are indications that certain animals, such as primates (Beran et al., 2016; Hampton et al., 2020), dogs (Fugazza et al., 2016, 2020), birds (Clayton and Dickinson, 1998; Raby and Clayton, 2012; Applegate and Aronov, 2022), and also rodents (Fellini and Morellini, 2013; Panoz-Brown et al., 2018; Sato, 2021), may have the capacity for episodic-like memory, which involves remembering specific events and their context, but may not include the subjective experience or self-awareness associated with human episodic memory. For example, studies have shown that chimpanzees (Martin-Ordas et al., 2010) and scrub jays (Clayton and Dickinson, 1998), can remember what they have seen, where they saw it, and when they saw it, which suggests they may be capable of episodic-like memory.

Mice are capable of recognizing objects and the location they have encountered them in before, which is a hallmark of episodic memory (Dere et al., 2008). However, it is important to note that the extent to which these abilities represent true episodic memory in the way that humans experience it is still a matter of debate. Many researchers believe that the cognitive processes underlying episodic memory in humans are much more complex than those in other animals, and that there may be fundamental differences in the way that humans and other animals remember events from the past. Overall, while mice may possess some forms of episodic-like memory, the question of whether they have true episodic memory remains an active area of research in the field of neuroscience.

### Conventional behavioral tests for the assessment of memory processes in mice

#### Object recognition test/novel object test

The ‘object recognition test’ is among the oldest and most widely used memory tests in rodents. The paradigm utilizes the natural tendency of mice to explore new/unfamiliar objects (Berlyne, 1950). The test itself is usually performed in two sessions, separated by an interval of variable duration between the sessions. In the first session (habituation session), the animal can explore two similar objects and in the second session (test session), one of the objects is replaced by a new, unknown object. If the mice are able to remember the familiar object, they spend more time with the new object. Depending on the length of the inter-session interval, either short-term or long-term memory can be investigated (Leger et al., 2013).

The object recognition test is used to assess the ability to recognize a familiar object, which is generally considered a form of declarative memory. Since the object recognition test primarily measures the ability to recall and recognize specific objects rather than the context in which they were encountered, the test is generally considered a test of semantic memory rather than episodic memory. The neuroanatomical structures involved in recognition memory include the hippocampus which is involved in the encoding and retrieval of spatial, semantic, and episodic memories. Also, the prefrontal cortex, critical for a wide range of visual behaviors, future planning, and impulse control is likely to be crucially involved. While the object recognition test is generally easy to set up and interpret, there are also some potential disadvantages that should be considered. As the object recognition test measures spontaneous behavior, a low trial count is inherent to the design, which is prone to increase variance across experiments (Ameen-Ali et al., 2015). Further, a lack of standardization of test objects may elicit diverging reactions from the animals, as a mouse for instance may display different reactions toward a climbable versus a chewable object (Dere et al., 2007). However, some protocols have been developed which mitigate some of those limitations (Leger et al., 2013; Wooden et al., 2021).

#### Spatial memory tasks

Spatial memory is crucial for any animal to navigate their environment and perform vital functions such as finding food sources or shelter. Typically, spatial memory is considered a subtype of episodic memory because it stores information within the spatio-temporal frame (Keefe and Nadel, 1978; Sharma et al., 2010). There are several tests used to study spatial memory in mice. Various forms of mazes are often used.

A commonly used test for spatial learning and memory in mice is the Morris water maze (Morris, 1981). The test was initially developed for rats which, as opposed to mice, do not have a natural aversion to swimming. The mouse variant of the Morris water maze takes advantage of the species’ inherent aversion to swimming for additional motivation.

There are several protocols (e.g., Vorhees and Williams, 2006), all of which have in common that mice are placed in a circular pool of water and have to locate a platform just below the surface, which is not visible from the subject’s perspective. Visual cues are placed around the pool for orientation. During the test, the animal’s movements are tracked, and various measures such as time to find the platform, distance traveled, and swimming path are used to assess spatial learning and memory functions. The test is typically conducted over a period of several days, with the animal’s performance improving over time as it learns the location of the platform. The Morris water maze test is widely used in neuroscience research and considered to be a reliable method for assessing spatial learning and memory in rodents.

The test has been criticized for being stressful for the mice (Harrison et al., 2009). In addition, translational significance can be debated, as the test captures learning and memory functions in an extreme stress-situation. However, it is known that memory retention is enhanced after stress (Roozendaal and McGaugh, 1996; Barsegyan et al., 2010), whereas memory retrieval is impaired under stress (De Quervain et al., 1998; Cai et al., 2006). Interestingly, recurrent stress across life has been shown to improve cognitive performance in individual rats (Hadar et al., 2019).

A ‘dry’ version of the water maze test is the Barnes maze (Barnes, 1979), likewise originally performed in rats. Sixteen years later, the test was adapted for mice (Bach et al., 1995). The mice are placed on a raised platform with several holes around the edge of the platform. One of these holes is connected by a tube to the home cage or to a shelter box. In the Banes maze test, the natural aversion to brightly lit open spaces is used to motivate the mice to find the correct hole to escape from the platform. Visual cues are also placed around the platform to test special learning and memory. Interestingly, also the Barnes maze test elicits a significant increase in stress hormones, especially in the first days of training (Karabeg et al., 2013). However, it has been shown that 30 min after the final trial glucocorticoid levels are lower in mice tested in the Barnes maze than in mice being exposed to the water maze (Harrison et al., 2009).

Alternating behavior is well studied in the T- or Y-maze (Richman et al., 1986; Deacon and Rawlins, 2006; D’Isa et al., 2021). A reward is placed in one or both arms of the maze and the frequency and order of visits to each arm is measured. For spatial orientation, visual cues can be placed both inside and outside the maze. However, the T-maze is not suitable for investigating preferences for different items such as food rewards. While mice are able to navigate the maze and find rewards, they tend to display alternating behavior between the respective arms regardless of bait quality (Habedank et al., 2021).

To test episodic memory, a circadian-based time-place learning task has been developed in a three-armed maze. The animals have to learn to associate a stimulus with a place and time of day (Van der Zee et al., 2008; DeVito and Eichenbaum, 2010; Mulder et al., 2013, 2015). This is done by creating a time- and place-dependent conflict between reward and punishment. The test is conducted at three separate times of the day. All three arms are baited, but at various times of the day, one arm is additionally punished with a foot shock. The animals have to learn which arm to avoid at the respective times of day in order to evade the punishment.

#### Conditional learning paradigms

In conditioned learning tasks, presented stimuli are associated with specific outcomes or rewards. Animals are typically trained to perform a specific task or show a behavioral reaction in response to the stimulus.

A prominent example for conditional learning is operant conditioning. Operant conditioning involves learning a stimulus–response pattern from spontaneous behavior. Experiments based on the Skinner box paradigm (Skinner, 1935) are used to train mice to perform wanted behavior or omit unwanted behaviors. This type of experiment deliberately reduces the number of potentially distracting stimuli. Operant conditioning changes the frequency of a behavior that is spontaneously shown. In mice, this can be pressing a lever (Jurado-Parras et al., 2013; Lintas et al., 2021), performing a nose poke (Krackow et al., 2010; Kahnau et al., 2023b), or touching a touch screen (Krakenberg et al., 2019). By providing a reward, the frequency of lever pressing, or nose poking can be increased. Vice versa, a punishment will decrease the frequency of a behavior. The operant behavior can additionally be associated with external stimuli such as sounds (Chen and De Hoz, 2023; Kahnau et al., 2023b), which are presented depending on the change in behavior to be trained.

In shuttle box experiments, the mice are additionally conditioned to different stimuli such as light or tones (Clark et al., 2003; Moragrega et al., 2005). The shuttle box is divided into two equal compartments with a barrier between them. The animals have to learn to either switch compartments or remain, respectively, once a stimulus occurs in order to avoid a foot shock punishment. This type of box requires the mice to learn and remember sensorimotor associations.

The conditioning place preference test utilizes the principle of classical conditioning (Bardo and Bevins, 2000). Neutral stimuli (e.g., visual cues) are associated with an unconditioned, motivationally significant stimulus (e.g., food). After successful conditioning, the formerly neutral stimuli elicit similar responses to the unconditioned, motivational significant stimulus and thus the neutral stimulus becomes a conditioned stimulus. In the conditioning place preference experiment itself, the mice are then placed in a test apparatus consisting of two equally sized compartments, with each containing a conditioned stimulus. A preference test then measures how much time the mice spend in each compartment. In this way, the valence of the unconditioned stimuli is examined by preference for the respective conditioned stimuli.

#### Limitations of conventional behavioral tests

The above discussed conventional learning- and memory tests share the following qualities: (1) they are performed in an experimental apparatus distinct from the animals’ home cage, to which the animals need to be transferred and habituated for experimental procedures, (2) animals are tested individually (or dyadically), and (3) they require extensive experimenter intervention for experimental protocols and data recording. Inherent to these shared characteristics are a few caveats: experimental procedures using external apparatuses such as mazes are time-consuming to set up and with regard to habituating the animals (Vorhees and Williams, 2006; Leger et al., 2013; Willis et al., 2017). Additionally, there usually is a stark contrast between the time demand on the experimenter for carrying out the experiment and the actual experimental time recorded (usually in the order of minutes). The time of experimentation also rarely coincides with the rodents’ circadian period of activity (which is the night in nocturnal rodents such as mice). Deviance from those phases have been found to increase animal stress and are suited to confound results (Roedel et al., 2006).

Commonly used spatial learning tests such as the Morris water maze or the Barnes maze utilize stress as a motivator for the participation of the animal in the experiment (exposing the animal to water in case of the Morris water maze; brightly lit open arenas in case of the Barnes maze). Yet, learning success in such experiments has been found to be inversely correlated to animal stress levels during the trial (Harrison et al., 2009). Even when stressors are not part of the experimental design, removing a mouse from its familiar environment and social group will always elevate stress levels and in turn potentially confound results (Krohn et al., 2006; Manouze et al., 2019). Freedom from pain and stress reduction in behavioral tests are essential both for animal welfare and for reproducibility of experimental results, and these conditions should be avoided as much as possible, unless they are indispensable for experimental reasons. This applies in particular to learning and memory tests, as these are particularly susceptible to stress (de Quervain et al., 1998; Cai et al., 2006). Further, behavioral experiments involving animals are particularly prone to be impacted by the experimenter effect (interpersonal variance dependent on experimenter), as even if multiple persons were to replicate experimental procedures perfectly, mice would still display plasticity in their reactions toward the experimenter based on factors such as experimenter sex, smell, etc. (Bohlen et al., 2014).

Many conventional learning tests for mice are based on an artificial and simplistic design derived from an anthropocentric understanding of cognition and intelligence. Therefore, these test environments do not usually reflect real-life situations and are consequently lacking in ecological validity. Ecological validity is considered a form of external validity that refers to whether the research reflects real, naturalistic conditions (Parsons et al., 2023). In animal experimental research, ecological validity often takes a back seat in favor of better control of experimental conditions (Kondrakiewicz et al., 2019). To overcome this, test systems for measuring learning and memory performance in laboratory animals should use naturalistic situations with relevant test settings that correspond to naturally occurring contexts relevant to the species being tested.

Recently, two trends in refining memory tasks have become apparent: for once, researchers started to shift the location of memory testing from external apparatuses such as mazes and arenas to the animals’ familiar environments. Often, the trials take place either in the home cage, or in areas the mice may access from their home cage. This does not only alleviate the time required to habituate the animals to the testing areas, but also reduces the amount of stress exerted on the animal through handling. In cases where the trialing apparatus can be accessed voluntarily, the agency on the test subject’s side is additionally increased, allowing for the trials to take place during the rodents’ natural time period of peak activity and high motivation.

Secondly, advances in computer vision and machine learning allow for a greater degree in automation in experiments that demand extensive monitoring. This does not only open new ways of acquiring and interpreting data, but also is suited to reduce the experimenter effect during trialing. Taken together, those developments may pave the road for new methods in memory testing in mice, which will be shed light on in the next chapters of this review.

### Tests beyond the mainstream

#### Home cage-based learning and memory tasks

Several test systems have been developed to study spontaneous behavior as well as learning and memory of mice in their home cage environment. Home cage systems offer the possibility of testing laboratory animals in their familiar environment and undisturbed by the experimenter.

The use of such home cage systems has increased in the past (Kahnau et al., 2023c). This development spawned movements such as the COST Action initiative “Improving biomedical research by automated behavior monitoring in the animal home-cage”,^1^ which was established in 2021. This initiative unites scientists from all over Europe using, developing, as well as educating about different home cage-based systems.

One such system is the PhenoTyper (Noldus), which, in addition to measurements of spontaneous activity and circadian rhythm, also allows for operant conditioning experiments. The system houses food, water, a reward dispenser, a two-entry shelter, LEDs, a video camera and, if necessary, a three-entry CognitionWall. Mice can be conditioned to receive a food reward when they enter the shelter or CognitionWall through a defined hole (Maroteaux et al., 2012; Remmelink et al., 2016). Successful adaptation to changes in the environment sometimes requires discarding or modifying learned behaviors, respectively. With the PhenoTyper it is possible to test flexibility by setting another entry of the shelter or CognitionWall as correct and rewarded. Other studies deployed the home cage environment to condition mice to auditory stimuli (Francis and Kanold, 2017; Francis et al., 2019; Alipio et al., 2021). Mice were conditioned to different tone frequencies in go/no-go discrimination tasks. For the correct response to one stimulus, the mice received water as a reward; for the response to the other stimulus, the mice received a punishment. By installing so-called ‘add-ons’ such as different wall types, a feeding monitor, or the PhenoWheel, the PhenoTyper system may be customized to be equipped for a rather diverse set of research questions. Those ‘add-ons’ however must be purchased individually, which can turn setting up a potent PhenoTyper system into a costly endeavor.^2^

The disadvantage of the previously described systems is that although the mice can be housed in groups, individual housing is required to record individual data. To collect individual data from mice kept in groups, RFID (Radio Frequency Identification) systems can be used. RFID antennas record the activity through transponders which are usually subcutaneously implanted into the neck region of the mice.

**Figure 2:**
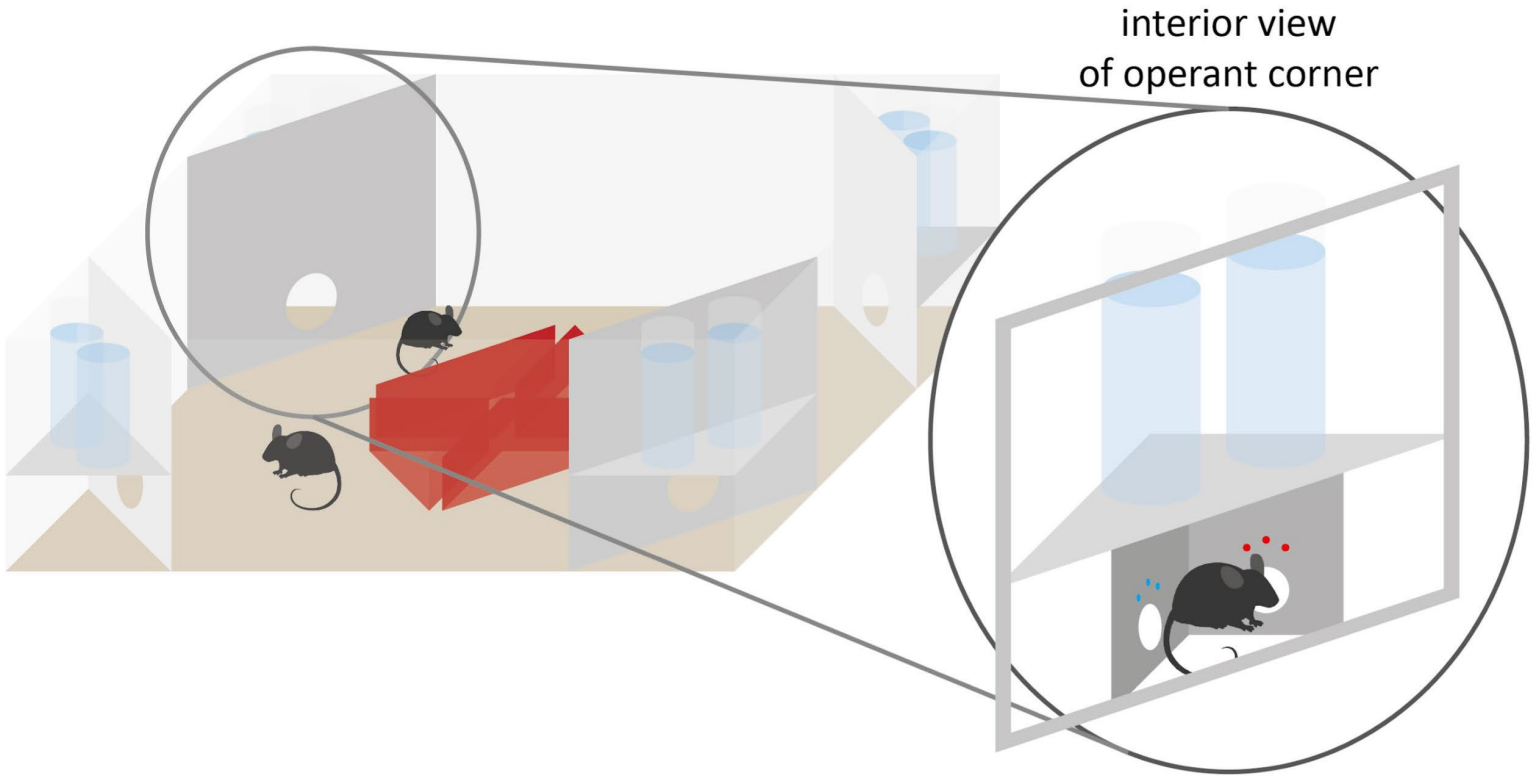
IntelliCage schematic with operant corner. Each operant corner houses two doors through which conditional access to reward liquids can be granted to individual animals. The corner further allows to punish the animals via airpuffs and to visually signal to them via tricolored LEDs.

One RFID-based system is the IntelliCage (IC, Figure 2). The IC itself serves as a home cage and enables the testing of learning and memory and has already been used in several studies (e.g., Krackow et al., 2010; Endo et al., 2011; Kiryk et al., 2020; Kahnau et al., 2021). The IC is embedded in a type IV cage and contains four independent conditioning corners. Up to 16 mice can be housed as a social group. Each corner is equipped with an RFID antenna. Aided by additional presence sensors within the corners, the identities of the mice frequenting the corners are determined via the RFID transponders as well as metrics of the individual visits such as time and durations. Only one mouse can be in one corner at a time. The corners also provide access to two independent water bottles. Access can be granted or denied via doors. Above each door three LEDs are positioned to provide additional cues. There is also an infrared nose poke sensor on each door. The system can be set that the doors are always open, open once a corner is entered, or open only when nose pokes are performed on the nose poke sensor. There is also an air valve in each corner to punish any error (e.g., entering a wrong corner or nose poke at a wrong nose poke sensor) with an airpuff (Voikar et al., 2018; Iman et al., 2021; Kahnau et al., 2021).

The IC system is based on the concept of using water (or other liquids) as a reward. With the RFID system it is possible to give each mouse individual access to the liquids according to certain patterns. The animals have to learn in which corner they can access the liquid.

The IC has been used in many studies with different learning paradigms. A supposedly simple learning experiment within the IC is the place learning experiment, which can be used to investigate spatial short-term memory (Mechan et al., 2009; Krackow et al., 2010; Voikar et al., 2018; Kahnau et al., 2021). Each mouse is assigned one of the four corners as the correct corner. Only in this corner the mice gain access to water by making a nose poke at the nose poke sensors to open the doors. Compared to the Water maze or Barnes maze, which also probe spatial memory, the mice in the IC do not have to be removed from their familiar environment and separated from the social group for carrying out the testing. However, the space in the IC is comparatively small and the animals’ motivation to run and explore is relatively high, which can lead to higher error rates and thus misinterpretation of learning behavior (Voikar et al., 2018). Nevertheless, it has been shown that mice which have a lower escape time in the Water maze also show fewer errors in the IC place learning task (Lipp et al., 2005).

By defining not only one corner as the correct corner, but different corners as well as their alternation, temporal learning, place orientation and behavioral flexibility can be studied in addition to spatial memory (Krackow et al., 2010; Endo et al., 2011).

In the behavioral sequencing paradigm, diagonally opposite corners are alternatingly assigned as ‘correct’ so that after a correct visit, the animal then needs to visit the opposite corner. In serial reversal learning, two operant corners are initially assigned as ‘correct’ and after a period of time, those switch so that the two previously inactive corners become the subsequently correct corners. This prevents individual mice from concentrating on certain corners that are close together, which could lead to misinterpretation of the animals’ learning behavior.

Episodic memory can be tested by adding drinking phases, similar to the three-armed maze. The mice not only have to learn which corner currently grants access to water, demanding them to remember which corner they were in before, but also at which times the doors in the correct corners can be opened by nose poking (Voikar et al., 2018). One limitation of the system, however, is that only liquid rewards can be presented to the animals by virtue of its design.

Another limitation of the IC is that, depending on the size of the group, one corner may be simultaneously assigned as correct to multiple animals. As a result, it may occur that a mouse, despite successfully learning the time and location of an accessible corner, finds it occupied by another mouse. This can result in a different and thereby incorrect corner being visited. This in turn will lead to a false increase in the number of errors and give a false picture of the learning success of the mice.

A way to avoid this is to connect the IC as a testing arena to a standard home cage via a gate (AnimalGate; TSE). The doors of the animal gate, regulated by RFID and infrared sensors, allow only one mouse at a time to pass from the home cage to the IC. This allows individual mice to perform different tasks undisturbed by group members. Kahnau and colleagues used this setup to condition mice to perform a daily increasing number of nose pokes in order to access different liquid rewards, as well as to condition mice to different tone frequencies (Kahnau et al., 2023a).

Winter and Schaefers developed a gate system (IDsorter; PhenoSys) which allows mice to move individually between the home- and the test cage similarly to the TSE AnimalGate (Winter and Schaefers, 2011). In further studies, an eight-armed maze was connected to the home cage, allowing spatial learning and memory to be studied in a home cage-based manner (Mei et al., 2020; Kohler et al., 2022). Connecting experimental apparatuses to animals’ home cages via gates that can be passed by the rodents voluntarily allows to perform studies relying on contraptions not incorporable into a home cage setting. At the same time, a gate avoids having to take subjects out of their familiar environment and group for the duration of the test.

#### Sequential problem-solving: lockboxes

Based on Thorndike’s puzzle box for cats (Burnham, 1972), attempts were made to investigate innovation using multistep problems in other species such as great apes (Whiten et al., 1996; Carvalho et al., 2008, 2009), corvids (Hunt, 1996; Taylor et al., 2007; Bird and Emery, 2009; Wimpenny et al., 2009; Cheke et al., 2011), keas (Huber et al., 2001; Miyata et al., 2011), pigeons (Epstein et al., 1984), and cockatoos (Auersperg et al., 2013), which may also give hints about insight learning. Examples for multi-step problems are so-called lockboxes that consist of different mechanisms (e.g., screws, bolts, levers, wheels) blocking each other (Auersperg et al., 2013). To access a food reward, the locks have to be opened in the correct order.

Haptic exploratory behaviors associated with intense manipulation appear to be beneficial for solving lockboxes when compared to visual exploration (Auersperg et al., 2013). However, the most efficient solution to a problem may not be identified at the first attempt by haptic exploration, but can be found when the lockbox has been manipulated for a while (Auersperg, 2015). Animals able to solve the different mechanisms of a lockbox and replicate the solution path seem to possess behavioral flexibility, sensorimotor control, and procedural memory (Auersperg et al., 2013). Behavioral flexibility is defined as “behavioral adjustments in response to external or internal stimuli” (Strier, 2022), which requires the animal to be innovative, learn from consequences, and display inhibitory control (Griffin and Guez, 2014; Daniels et al., 2019). Multi-access lockboxes, that can be opened in multiple ways, allow blocking a solution and monitoring whether the animals can find an alternative solution (Auersperg et al., 2011; Daniels et al., 2019). In raccoons, the use of multi-access lockboxes revealed that neophobia, persistence, and the diversity of behaviors shown while interacting with the lockbox were predictors of the animals’ performance in solving the problems (Daniels et al., 2019).

In studies involving cockatoos, some individuals showed sensitivity toward the blocking effect of the locks: they did not entirely remove the blocking mechanisms, but just enough to move the next mechanism. Auersperg et al. (2013) concluded that cockatoos learned to open the lockboxes by combining exploratory behavior, learning from consequences of their actions, and goal directedness.

Animals may succeed in solving multi-step lockboxes by understanding the physics underlying the lockboxes and the physical causal connection of the different mechanisms. Since lockboxes require an animal to deal with multiple problems/steps (i.e., means) and execute a sequence of (planned) actions to achieve a goal (i.e., end), they can be considered as means-means-end problems, comparable to using a sequence of tools (Santos et al., 2005; Auersperg et al., 2013). When an animal opens a lockbox for the first time, the information may be saved as part of the episodic memory but may be transferred to the semantic memory if this experience is repeated several times. However, associative learning (operant conditioning) can also play a major role with regard to sequential problem-solving: the animals can learn to solve the lockboxes if (1) each step is reinforced (Epstein et al., 1984), or (2), the locks are stepwise added from the food reward in reciprocal order (i.e., a lock closer to the food reward can serve as secondary reinforcement for the opening of a more distal lock located; Auersperg et al., 2013), or (3) the food reward is represented (i.e., stepwise removal of the distal locks and getting closer to the food reward may be rewarding itself; Auersperg et al., 2013).

After an animal has had the chance to extensively explore a problem, transfer tasks can be performed to distinguish whether it only displays a sequence of reinforced behaviors or considers the physics underlying the problem (Santos et al., 2005; Auersperg et al., 2013; Auersperg, 2015). For instance, if the first, second, and fourth lock of a 4-step lockbox (with the first lock being the closest to the goal) are closed, but the third lock is open, a subject that gained a deeper understanding of the entire sequence of mechanisms will omit the irrelevant locks (i.e., the fourth and third lock) and will mainly manipulate the second followed by the first lock.

**Figure 3:**
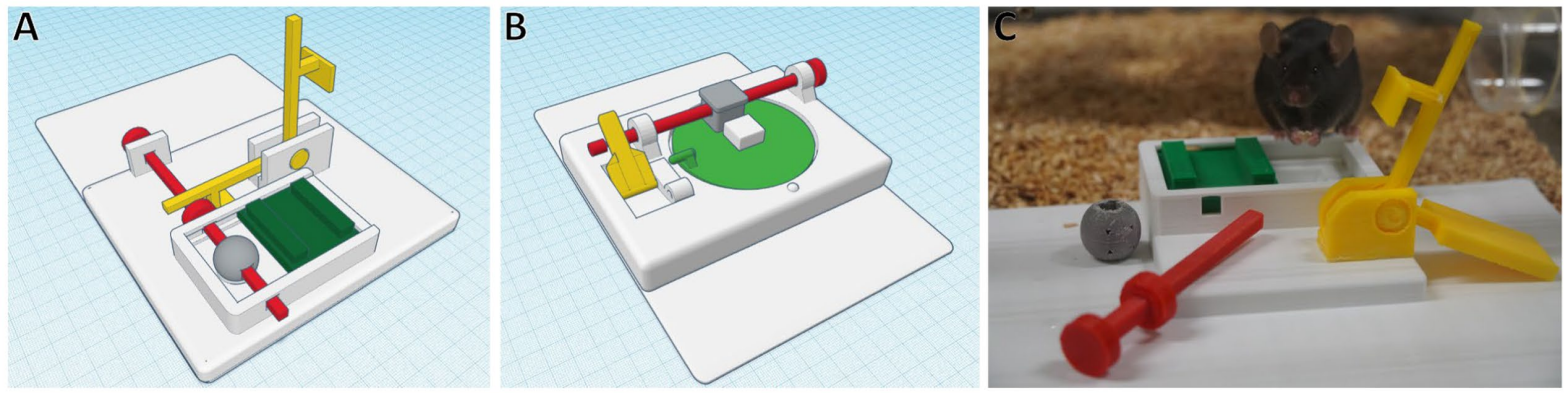
Four-step lockboxes. Food rewards are hidden and blocked by the green elements, which are again blocked by a chain of interconnected mechanisms. To solve the mechanism, the mouse needs to (1) flip the yellow lever, (2) pull the red stick, (3) remove the gray ball/cube and (4) move the green door to uncover the reward. Parts can be created using a 3D printer. Each lockbox has a size of about 22 cm x 16 cm. **(A)** Lockbox with sliding door **(B)** Lockbox with rotating disk **(C)** C57BL6/J mouse with opened 3D-printed lockbox **(A)**. Photograph by Katharina Hohlbaum.

For mice, the most common animal species used in research, the authors of the present review developed two lockbox sets (Figure 3), each consisting of four 1-step and a 4-step lockbox combining the four single mechanisms.^3^ To open the lockboxes, the mice have to display different behaviors, i.e., lift a lever, pull a stick, carry a ball/cube away, push a sliding door or rotate a disk. A deep learning-based computer vision pipeline for the automated analysis of the animals’ interactions with the lockboxes and their learning progress is currently being developed by the authors of the present review and their collaborators.

Vrbanec et al. introduced a series of simple puzzles in order to investigate whether the ability of problem-solving of wild house mice (*Mus musculus*) improved as an adaptation to the urban environments (Vrbanec et al., 2021). These simple puzzles required the mice to move a sliding lid, lift a petri dish, remove paper from a tube, open a small window, carry a lid away, or tilt open a lid. In a simple 2-step puzzle, the reward was hidden in a tube that was blocked with paper and placed vertically in a cage. These tasks were also used to compare the innovation in striped field mice (*Apodemus agrarius*) from rural and urban environments, respectively (Mazza and Guenther, 2021).

In earlier years, a puzzle box for mice that is more reminiscent of Thorndike’s puzzle box, i.e., tasking mice with escaping from an aversive environment, was introduced for screening executive functions both in general and in particular for schizophrenia mouse models. This puzzle box is an arena that is divided into a brightly lit start and a smaller, covered goal compartment. The latter can be entered through a narrow, obstructed underpass after the mouse removes the obstructions by digging, climbing, pushing, manipulating, and removing objects (Galsworthy et al., 2005; Ben Abdallah et al., 2011).

Overall, this approach represents a promising paradigm for understanding higher cognitive functions in model animals–especially as improved, deep learning-based observational methods will enable quantitative data acquisition.

## Learning in social interaction

The wild-dwelling relatives of the laboratory mouse (*Mus musculus*) aggregate in large social groups (Berry, 1970), forming territories and social hierarchies (Mackintosh, 1970; Mondragón et al., 1987). This highly social lifestyle entailed the evolution of means for social transmission of information and learning from social interactions. Those in turn are utilized in biomedical research, principally for the identification of impairments of social behavior as they may arise in transgenic autism models or novel compound testing (Moy and Nadler, 2008; Silverman et al., 2013). Most prevalent are assays of social amnesia and social novelty in dyadic or triadic interactions. Classically, those interactions take place in a separate experimental area distinct from the animals’ home cage and are temporally limited (in the order of minutes; Stevenson and Caldwell, 2014).

Common to those tests is the underlying principle of neurologically unimpaired rodents generally displaying greater interest toward novel social stimuli over familiar ones (which is the same principle that is used in the object recognition test/novel object test described previously), which manifests in prolonged exploration behaviors (approaching, nose-to-nose or nose-to-anogenital sniffing, following, grooming; Lee et al., 2018). Tests of social memory retention usually involve the habituation of the subject to an previously unfamiliar ‘intruder’ (directly or via olfactory cues; Oettl et al., 2016) over either one or multiple sessions (Ferguson et al., 2000). Then, an entirely novel conspecific is introduced, presented either subsequent to or in conjunction (‘Three-chambered social memory test’; Molosh et al., 2014) with the previously familiarized animal. A relatively decreased interest in the novel stimulus is interpreted as impaired memory of social interactions. Social memory is regulated by ventral hippocampal projections to the medial prefrontal cortex (Phillips et al., 2019) as well by the amygdala, where oxytocin plays a crucial role in acquiring social memories (Ferguson et al., 2000).

The value of dyadic social memory- and social novelty tests for identifying deficits in social behavior is well established. However, awareness of their limitations is essential. Those assays were designed to examine merely a singular aspect of the murine social behavioral repertoire–affinity and aversion toward novel social stimuli, respectively–and they cannot offer much insight into an animal’s social capabilities and limitations beyond. To phenotype a new transgenic strain or chart models of neurodevelopmental disorders associated with impairment of social interaction, more extensive testing is recommended. The complexity of murine social behavior allows for more in-depth investigations.

Olfactory cues are counted as the greatest determiners of the outcomes of social interactions between mice (Smotherman et al., 1974). The ‘Social transmission of food preference test’ (Strupp and Levitsky, 1984; Valsecchi and Galef, 1989) elegantly demonstrates learning of olfactory cues. In this paradigm, a ‘demonstrator’ mouse is fed a distinctly scented (usually cinnamon- or cocoa-laced; Kogan et al., 1997) food and is subsequently allowed to interact with an ‘observer’ conspecific. When the observer is later presented with a choice between the cued and a novel scented food, unimpaired rodents will display a strong preference for the option the demonstrator consumed (Wrenn, 2004). A divergent choice may indicate impaired olfactory memory or disturbed social transmission. Studying the role of olfactory cues in rodent communication and social behaviors is limited by insufficient technological means to represent odor trails in the animals’ environment. Hence, their trajectories can only ever be measured indirectly through the animals’ behavior (Zou et al., 2015).

The ability of mice to learn from their conspecifics has been demonstrated to exceed olfactory cues. Another well-established paradigm for examining learning and retention of socially transmitted information is presented by ‘Social learning of fear’ (Jeon et al., 2010). The paradigm entails an observer being presented with a demonstrator’s unconditioned response to an unconditioned aversive stimulus, such as electric foot shocks (Guzmán et al., 2009). The observer displays an elevated fear response during the trial, but also when presented with the same experimental context in absence of aversive stimuli. The magnitude of the associated fear response is dependent on familiarity of the observer with the demonstrator (Jeon et al., 2010), suggesting the paradigm to not only offer a memory test for social cues, but further a possible model for empathy.

Kinship also enhanced social learning of predator-avoidance behavior in a study conducted by Kavaliers et al. (2005). Deer mice (*Peromyscus maniculatus*) which had previously observed conspecifics being bitten by micropredatory flies (*Stomoxys calcitrans*) displayed increased avoidance and analgesic responses when exposed to modified flies incapable of biting. As with other paradigms of associative fear learning (Jeon et al., 2010), avoidance and analgesic responses were correlated to kinship. Those observations suggest a general and considerable influence of subject familiarity on the outcome of memory tests involving social interactions, which is essential to be considered in selecting subject and stimulus animals for experiments. Sex (Valsecchi and Galef, 1989) and intragroup dominance status (Kavaliers et al., 2005; Williamson et al., 2016) have been found to impact social learning success in a similar fashion.

Observational learning of complex sequential behaviors exceeding socially transmitted preference and aversion (operant observational learning) is sparsely documented in mice. Comparatively few studies found observer mice to perform better in baited mechanical puzzles after demonstration by conspecifics (Mainardi et al., 1988; Carlier and Jamon, 2006). Such studies deployed a puzzle box usually requiring several steps to gain access to a food reward. Naïve observer mice are presented with the puzzle in presence of a trained demonstrator mouse, either allowing them to interact freely with the demonstrator (Valsecchi et al., 2002), or separated from them by a barrier only allowing for visual cues (Carlier and Jamon, 2006). These studies generally found observer mice to exhibit shorter latencies in solving puzzles tasks than controls, however none of them demonstrated true mimicry in the mice.

Of note, such complex mechanical puzzles are likely rather distant from any problems rodents face in the wild. While their validity as tools to test general cognition and problem-solving ability are widely accepted, the ability to learn from conspecifics through sequential mimicry may not have been strongly selected for in murine ancestors. Further, with olfactory cues and ultrasonic vocalizations (USVs) playing a pronounced role in murine communication (Portfors and Perkel, 2014; Ferhat et al., 2016), the importance of visual cues in social learning in mice remains unclear.

In recent years, efforts have been made to address some of the above issues by considering naturally occurring behaviors and the complex social structures of mice. This was preceded by the increasing availability of automation and animal tracking, which may also be applied for home cage monitoring. A selection of commonly applied automated tracking approaches is discussed in the next chapter of the review.

While ‘classic’ dyadic tests of social memory can offer only a very restricted slice of an animal’s actual social repertoire, continuous tracking over prolonged periods of time can paint a more conclusive picture. Thereby, subtle alterations in an animal’s social behavior may be recorded which would not have become apparent in a 10 min social interaction test.

Further, advancing automation and home cage-based monitoring allow for the gathering of data without direct interference of an experimenter, which has been shown to generate more reproducible results. The vast amount of data generated by modern machine-learning-supported investigations of rodent social interactions allow for more in-depth analyzes than were historically possible, but also pose new challenges to the researchers (for review, see Von Ziegler et al., 2021). The increase in variables comes with greater demands on computational power and expertise. In the age of big data and unsupervised machine-learning, a simple dyadic social approach test may still give the appropriate answer to a clear-cut research question. The future likely belongs to the automatons, but asking the right questions will remain the researcher’s responsibility.

### Automated tracking solutions

Subcutaneously implanting animals with RFID transponders has become a fairly standard practice, which is used in highly automated experimental setups for monitoring trials and controlling rewards, but also can be used for tracking the animals’ activity and association with cagemates. Additionally, owing to the recent advances in deep learning, computer vision-based automated pose estimation methods have become increasingly potent in quantifying animal behavior. Some of the most widely used solutions are DeepLabCut (Mathis et al., 2018) and LEAP (Pereira et al., 2019), which are based on supervised learning methods for continuous estimating poses of animals in single-camera 2-dimensional recordings or 3 in dimensions in case of multi-camera recordings (Nath et al., 2019; Mashall et al., 2020) for prolonged periods of time. These can be used to automate time consuming measurements (Sturman et al., 2020), for example of the time spent in a certain location (as in the Morris water maze or shuttle box experiments), the frequency of visits (as in the T-Maze), or the latency to solve a task (as in observational learning tasks). The recorded data is initially large in size and of high frequency, but the trackers reduce it to meaningful bits of information.

Most methods only require some initial human labor for generating an annotated dataset of images in which specific body parts of the animal are labeled. The annotated dataset then is used for training the algorithm. Other methods, such as SuperAnimal (Ye et al., 2023), do not even require any additional human labeling. Detecting multiple animals simultaneously in the case of social learning experiments has shown to be a challenging problem. Interactions between animals cause occlusions and the number of animals visible in an image might be unknown. If the number of animals is known, this information can be used to significantly enhance identification of individuals under conditions where occlusions occur (Dolokov et al., 2023). Some approaches such as the ‘Live Mouse Tracker’ (De Chaumont et al., 2019) or the ‘RFID-Assisted SocialScan’ (Peleh et al., 2019) combine RFID technology with machine-learning-mediated video tracking to mitigate either technology’s limitations. Other methods such as multi-animal DeepLabCut or SLEAP (Lauer et al., 2022; Pereira et al., 2022) make use of separate or integrated algorithms to accurately assign the detected limbs to the correct animals.

Automated methods for identification of behavioral hallmarks are capable of extracting individual and social behavioral features of animals, often performed in an unsupervised manner (Wiltschko et al., 2015; Segalin et al., 2021; Weinreb et al., 2023). In addition to an immense reduction of the manual labor required and the possibility to track and analyze animal behavior for longer periods of time, these methods are invariant to observer drift and might furthermore detect subtle behavioral hallmarks which would go undetected by a human annotator.

Many of the mentioned currently prominent solutions are still actively being developed together with the field as a whole. New software is released on a regular basis and new features are often added to existing software packages, so that the description of systems in a literature review will soon no longer reflect the current status.

## Conclusion

While the majority of laboratory mice are still housed in standard cages for testing in external apparatuses, a slight trend in the literature striving toward more naturalistic settings becomes apparent (Vyssotski et al., 2002; Hess et al., 2008; Lewejohann et al., 2009; Mieske et al., 2021). More and more studies deploy housing environments that provide more space, allow large groups to be housed, or provide conditions that approximate natural conditions. For laboratory experiments, alternative systems that allow maximum enrichment and housing of a large group of animals can promote natural and individual behavior in mice (Freund et al., 2013; Kempermann, 2019; Mieske et al., 2021). Interestingly, some researchers have drawn parallels between this approach and the methods of the pioneers of ethology, who conducted their studies by observing naturally occurring animal behavior in the wild (Smith, 2023). We believe that the turn toward naturally occurring behaviors in investigating learning and memory does not necessarily represent a rediscovery of lost scientific method. Rather, the advent of novel technologies provides the tools to expand the scope of “conventional” tests, sensibly analyze naturally occurring behaviors within laboratory settings and advance more ecologically valid behavioral assessment of learning and memory functions.

For the design of future experiments in general, and specifically in learning and memory, we suggest rethinking conventional approaches. By integrating testing apparatuses into the home cage or making them accessible from the home cage, the animal agency is maximized while any influence of the experimenter potentially impacting results is kept at a minimum. Those measures are suited to decrease animal stress levels during the experimental procedures (Krohn et al., 2006; Manouze et al., 2019) which in turn increases the reproducibility of memory tests (Strekalova and Steinbusch, 2010). Further, it fosters the goal of refinement in animal testing.

When combining home cage-based memory tests with novel machine-learning based solutions for tracking multiple animals over extended periods of time, powerful high-throughput setups for the generation of behavioral data are conceivable. By not limiting data acquisition to a snapshot of the animals’ behavior, such approaches may grant a deeper insight into memory functions that are hardly achievable by conventional tests. However, such systems are cost-intensive to set up and maintain and are inherently more demanding on computation and interpretation of results. As the discipline is still a very young one, it yet lacks standardized pipelines for data analysis that are universally and readily accessible. With the current exponential development in machine learning, and the growing social awareness for animal welfare and refinement of experimental procedures, we expect to see an increase in studies pursuing a minimally invasive, highly technologized approach to memory testing over the next decade. In this manner, with our incomplete understanding of learning and memory, we may yet build algorithms approximating artificial intelligence which in turn will help us understand the inner workings of biological intelligence.

## Author contributions

BL, PK, KH, PM, NA, MB, CT-R, LL, and KD contributed to the conception, literature search, and drafting and revising of the manuscript. All authors contributed to the article and approved the submitted version.

## Funding

This publication is based upon work from COST Action Teatime (CA20135), supported by COST (European Cooperation in Science and Technology). This work was funded under Germany’s Excellence Strategy–EXC 2002 “Science of Intelligence”–project number 390523135.

## Conflict of interest

The authors declare that the research was conducted in the absence of any commercial or financial relationships that could be construed as a potential conflict of interest.

## Publisher’s note

All claims expressed in this article are solely those of the authors and do not necessarily represent those of their affiliated organizations, or those of the publisher, the editors and the reviewers. Any product that may be evaluated in this article, or claim that may be made by its manufacturer, is not guaranteed or endorsed by the publisher.

## Footnotes

^1^TEATIME: https://www.cost-teatime.org/

^2^ https://www.noldus.com/phenotyper

^3^ https://github.com/RefinementReferenceCenter/MouseLockBox
